# Supplemental Fiber Affects Body Temperature and Fecal Metabolites but Not Respiratory Rate or Body Composition in Mid-Distance Training Sled Dogs

**DOI:** 10.3389/fvets.2021.639335

**Published:** 2021-04-29

**Authors:** Emma Thornton, Eve Robinson, James R. Templeman, Lindy Bruggink, Michael Bower, John P. Cant, Graham P. Holloway, Kelly S. Swanson, E. James Squires, Anna K. Shoveller

**Affiliations:** ^1^Department of Animal Biosciences, University of Guelph, Guelph, ON, Canada; ^2^emka TECHNOLOGIES Inc., Sterling, VA, United States; ^3^Department of Human Health and Nutritional Sciences, University of Guelph, Guelph, ON, Canada; ^4^Department of Animal Sciences, University of Illinois at Urbana-Champaign, Urbana, IL, United States

**Keywords:** physiology, canine, exercise, dietary fiber, nutrition

## Abstract

Dietary fiber affects canine physiology in many ways, such as increasing colonic absorption of water and improving gut health, both of which may positively impact exercise performance. The objectives of this study were to investigate the effects of increased dietary soluble fiber and incremental training on respiratory rate (RR), internal body temperature (BT), body composition, and fecal metabolites in mid-distance training sled dogs. Fourteen dogs (12 Siberian and 2 Alaskan Huskies) were blocked by age, sex, and body weight (BW) and then randomly allocated into one of two diet groups. Seven dogs were fed a dry extruded control diet (Ctl) with an insoluble:soluble fiber ratio of 4:1 (0.74% soluble fiber on a dry-matter basis), and seven dogs were fed a dry extruded treatment diet (Trt) with an insoluble:soluble fiber ratio of 3:1 (2.12% soluble fiber on a dry-matter basis). Fecal samples were taken once a week. All dogs underwent 9 weeks of incremental exercise conditioning where the running distance was designed to increase each week. Every 3 weeks, external telemetry equipment was used to non-invasively measure and record RR and internal BT at resting, working, and post-exercise recovery states. Body composition was measured on weeks −1 and 9 using quantitative magnetic resonance. Body composition, RR, BT, and fecal metabolites were analyzed using a mixed model with dog as a random effect and week and diet group as fixed effects. Dogs on Trt had lower working and post-exercise BT than Ctl (*P* < 0.05). In addition, Trt dogs had lower recovery BT at weeks 2 and 5 than Ctl dogs (*P* < 0.05). Treatment dogs had greater fecal short-chain fatty acid concentrations than Ctl (*P* < 0.05). Diet had no effect on RR or body composition (*P* > 0.10), but exercise resulted in an overall 7% increase in lean and 3.5% decrease in fat mass (*P* < 0.05). These data suggest that increasing dietary soluble fiber may positively influence BT and gut health; however, it has no effect on RR or body composition. Soluble fiber did not negatively impact any measures of overall health and performance and should be considered for use in performance dogs.

## Introduction

Exercise has the capacity to affect whole-body physiology; however, high-intensity endurance training, such as that experienced by sled dogs, can lead to gastrointestinal (GI) disturbances, heat stress, and possible dehydration (1, 2). Nutritional solutions, such as increased dietary soluble fiber inclusion, may support exercise performance through mitigation of these deleterious effects (3–6).

Dietary fiber can be divided into two categories based on its solubility in water [i.e., soluble and insoluble fiber; (7, 8)]. Soluble fibers typically have an increased extent of fermentation by GI microbes yielding short-chain fatty acids [SCFAs: mainly acetate, propionate, and butyrate; (7)]. SCFAs play a variety of physiological roles, including serving as an energy source for epithelial cells, regulating epithelial barrier integrity, supporting the immune system, and modulating inflammatory responses (9–11). In addition, SCFAs increase water absorption in the GI tract (12). As sled dogs primarily thermoregulate via respiratory evaporation (i.e., panting), water loss by way of salivation increases considerably during exercise leading to signs of hypertonic dehydration (13). Hypertonic dehydration can affect a variety of physiological outcomes, including lowering respiratory rate (RR) and increasing internal body temperature (BT) in dogs (14–16). As a BT exceeding 42°C can have serious physiological consequences in dogs (17), the ability to dissipate heat during exercise is key to minimizing deleterious outcomes and maintaining performance. Therefore, as SCFAs can increase water absorption in the colon of dogs, providing a diet with optimized soluble fiber may prevent dehydration and aid in heat dissipation that could influence RR and BT during exercise (18).

Soluble fiber may also impact body composition of actively training sled dogs. For example, sedentary humans who increased their soluble fiber intake had lower body weights (BWs), body mass index, and fat mass (FM) than those ingesting maltodextrin as a control (19). FM and lean body mass (LBM) are common measurements of body composition and have been reported to change with exercise (13, 20, 21). However, the effects of increased dietary soluble fiber on body composition in an exercising dog model have not yet been directly studied.

Therefore, the objective of this study was to investigate the effects of an increased soluble fiber diet and an incremental training regimen on the outcomes of RR, BT, body composition, and fecal metabolites in mid-distance training huskies. We hypothesized that increased soluble fiber supplementation would decrease exercise-induced internal BT and FM, increase LBM and fecal SCFAs, and have no effect on RR in actively training sled dogs.

## Materials and Methods

### Animals and Housing

The study was approved by the University of Guelph's Animal Care Committee (Animal Use Protocol #4008). Twelve client-owned domestic Siberian Huskies (8 females: 8 intact; 4 males: 1 intact and 3 neutered) and 2 Alaskan Huskies (2 neutered males) with an average age of 3.75 ± 2.7 years (mean ± SD) and BW of 21.54 ± 2.83 kg were used in the study. Dogs resided and trained at an off-site facility (Rajenn Siberian Huskies, Ayr, ON) that had been previously visited and approved by the University of Guelph's Animal Care Services. During the study, dogs were group-housed in free-run, outdoor kennels (3.5–80 m^2^) containing anywhere from 2 to 10 dogs each.

### Diets and Study Design

Dogs were blocked for age, sex, and BW before being randomly allocated into one of two diet groups: control (Ctl; *n* = 7; 3 females: 3 intact; 4 males: 1 intact and 3 neutered) or treatment (Trt; *n* = 7; 5 females: 5 intact; 2 males: 2 neutered). For 2 weeks prior to the study period, all dogs were acclimated to a dry extruded Ctl diet [Champion Petfoods Ltd., Morinville, AB; for ingredient and nutrient composition refer to (22)] that met or exceeded all National Research Council (23) and Association of American Feed Control Officials (24) nutrient recommendations for adult dogs at maintenance. During both the acclimation and study periods, dogs were consistently fed once daily at 15:00 h. Feed allowance was first determined using historical feeding records and the calculated metabolizable energy (ME) content of the diet. Body weight was measured at baseline and each week after, and diet intake was adjusted to maintain baseline BW. Dogs in the Ctl group were fed the Ctl diet with an insoluble:soluble fiber ratio of 4:1 [dry-matter basis: 4,074 kcal/kg ME, 94% dry matter (on an as-fed basis), 47% crude protein, 25% fat, and 0.74% soluble fiber], while Trt dogs were fed a dry extruded diet with an insoluble:soluble fiber ratio of 3:1 [dry-matter basis: 4,120 kcal/kg ME, 94% dry matter (on an as-fed basis), 47% crude protein, 26% fat, and 2.12% soluble fiber; Champion Petfoods Ltd., Morinville, AB; Table 1]. BioMOS^®^, a mannan-oligosaccharide (MOS)-derived strain of *Saccharomyces cerevisiae*, oat soluble fiber, flaxseed meal, *Yucca schidigera* extract, and chicory root were included as soluble fiber sources in the Trt diet, in place of pea starch in the Ctl diet. These ingredients were chosen and added with the objective of reaching an inclusion level previously reported to positively influence canine GI metabolites (9, 25–27), while yucca was added in the Trt diet to control fecal odor (28). Daily rations were mixed with 1 cup of water before feeding; diets were mixed for 10 min to allow for a homogenous mixture. At feeding, all dogs were tethered and individually fed to allow for monitoring of food consumption. Dogs were allowed 30 min to eat their allotted food, and any orts were weighed and recorded daily. Throughout the entirety of the study, dogs had *ad libitum* access to fresh water.

**Table 1:** Nutrient content and ingredient composition^a^ of the control and treatment diet on a dry-matter basis.

**Nutrient contents**	**Trt**	**Ctl**
Metabolizable energy, kcal/kg (calculated)^b^	4,120.15	4,074.35
DM^c^, %	94.15	94.15
CP, %	47.46	47.92
EE, %	25.82	24.61
TDF, %	8.28	5.52
SDF, %	2.12	0.74
IDF, %	6.16	4.67
Ash, %	8.52	9.08

a *Ingredient composition: pork meal, pea starch, low ash herring meal, fresh chicken, chicken fat, dried chicken, chicken meal, fresh pork, fresh pork organ blend, flaxseed meal, chicken and turkey giblets, oat soluble fiber, spray dried pork liver, pork pal (liquid), hydrolyzed poultry protein, herring oil, pork pal (dry), sodium chloride, dried kelp, choline chloride, chicory root, enticer, alpha-tocopherol, BioMOS^®^, natural antioxidant (liquid), thiamine, pantothenic acid, pyridoxine, potassium chloride, taurine, Yucca schidigera extract, Sel-Plex, zinc proteinate, natural antioxidant (dry), and copper proteinate. Adapted from Templeman et al. (22); ingredients in bold were only included in the Trt diet*.

b *Calculated metabolizable energy based on modified Atwater values*.

c *DM, dry matter; CP, crude protein; EE, ether extract; TDF, total dietary fiber; SDF, soluble dietary fiber; IDF, insoluble dietary fiber*.

### Exercise Regimen

A 9-week exercise regimen was implemented with exercise distance increasing incrementally throughout the trial period. The dogs were anticipated to run 6 km a day (4 days a week) at week 0 and reach 56 km a day (4 days a week) by week 8, but ambient conditions played a role in determining the actual daily run distance (RD; Table 2). Training consisted of all dogs running on a 14-dog gangline with staggered, pairwise groupings of Trt and Ctl dogs. The gangline was attached to an all-terrain vehicle with one rider who controlled the machine in its lowest gear. A pace of ~20 km/h was maintained throughout the training period. Running speed and distance were measured using a speedometer and odometer, respectively, on the all-terrain vehicle.

**Table 2:** Mean daily run distance, feed intake, and BW data for all dogs from weeks −1 (exercise challenge distance) to 8, body composition data (lean body mass, fat mass, and total body water) at weeks −1 and week 8, and anticipated run distance for the proposed exercise regimen with mean temperature and humidity for each week.

	**Week**											***P*****-value**		
	**−1**	**0**	**1**	**2**	**3**	**4**	**5**	**6**	**7**	**8**	**SEM**	**DG^1^**	**Wk**	**DG × Wk^2^**
RD, km/day^3^	3.0^f^	5.8^d^^e^	11.2^c^	17.0^d^^e^	23.8^e^	30.1^d^	25.4^c^	20.4^b^	27.0^a^	37.1^a^	1.08	0.99	<0.01	1.00
ARD, km/day^4^	3	6	12	18	24	30	36	42	48	54				
FI, g/d	273.4^c^	325.9^d^	367.5^d^	427.3^c^	480.6^b^	570.9^a^	563.9^a^	560.4^a^	560.4^a^	560.4^a^	30.9	0.44	<0.01	0.93
BW, kg	21.7^a^^b^^c^	21.3^b^^c^	21.3^b^^c^	21.1^c^	21.0^c^	21.7^a^^b^^c^	22.1^a^^b^	22.1^a^^b^	22.3^a^	22.5^a^	0.34	0.34	<0.01	0.57
LBM, %	73.1^b^									80.1^a^	2.80	0.97	<0.01	0.92
FM, %	16.2^a^									12.7^b^	1.90	0.85	<0.01	0.15
TBW, %	54.7^b^									60.0^a^	2.02	0.96	<0.01	0.69
T, °C	−3.8^d^^c^	2.2^a^	2.4^a^	−2.0^c^^d^	0.1^b^	−7.8^f^	−0.6^b^^c^	2.3^a^	−6.0^c^^f^	−3.5^d^	0.6	0.82	<0.01	1.00
H, %	81.4^e^	95.0^a^	89.7^a^^b^^c^	86.2^bcde^	84.8^c^^d^^e^	89.3^a^^b^^c^	87.4^b^^c^^d^	91.0^a^^b^	80.7^d^^e^	81.3^e^	1.6	0.89	<0.01	1.00

*FI, feed intake; BW, body weight; LBM, lean body mass; FM, fat mass; TBW, total body water; T, ambient temperature; H, humidity*.

1 *DG, diet group; Trt, treatment (2.12% soluble fiber); Ctl, control (0.74% soluble fiber)*.

2 *Interaction effect between DG and Wk (week)*.

3 *RD, mean daily run distance determined as an average of the 4 days of each week the dogs trained*.

4 *ARD, anticipated run distance for proposed incremental exercise regimen (distance proposed to be run 4 days/week of training)*.

a−f *Values in a row with different superscripts differ (P < 0.05)*.

### Exercise Challenges

On weeks −1, 2, 5, and 8, one off-day in the dogs' training schedule (no running) was replaced by an exercise challenge. During each exercise challenge, dogs were run for 3 km at a pace of 20 km/h in either a three- or four-dog team. The distance run during exercise challenges was based on a previous study from our laboratory (29) as well as trail access and availability. Each group contained at least one Trt and one Ctl dog. Following the 3-km run, dogs were watered immediately. During each exercise challenge, all dogs were equipped with external telemetry jackets to non-invasively record both RR and BT (emka TECHNOLOGIES, Sterling, VA, USA).

### Telemetry Jackets

Collection of RR data was based on a previous study by Thornton et al. (29).

A novel pilot procedure to take non-invasive BT measurements was executed by modifying a commercial resistance temperature detector (RTD)-based skin temperature probe (emka TECHNOLOGIES, Inc.) into a hermetically sealed RTD-based rectal temperature sensor. The probes were lubricated and inserted ~2 in. into the rectum of two dogs per group. The probes were fixed to the base of the tail using adhesive tape and vet wrap. To validate the manufacturers' calibration, each probe was individually calibrated in water baths at 0 and 40°C. A standard laboratory-grade glass thermometer was used as a reference standard. Calibration was performed just prior to placement of the sensor. Internal BT was measured every second at resting (rBT), working (wBT), and post-exercise state (post-BT). Once the challenge ceased, dogs were immediately watered and remained on the gangline for 30 min to continue gathering RR and BT data until post-RR and post-BT were obtained.

### Blood Sample Collection and Analysis

Blood samples were collected and analyzed as described by Templeman et al. (22). In brief, fasting blood samples were collected on weeks −1, 2, 5, and 8 to assess standard serum veterinary diagnostic measurements and markers of nutritional and health status (Supplementary Tables 1, 2). Dogs were fasted for 12 h overnight, and 5-ml samples were collected by way of cephalic venipuncture with a serum Vacutainer^®^ system (Becton, Dickinson and Company, Franklin Lakes, NJ, USA). Whole blood samples (1 ml) were kept on ice prior to being analyzed for hematological indices [e.g., complete blood cell count (CBC)] using a Siemens ADVIA 2120 hematology analyzer (Siemens Healthcare Ltd., Oakville, ON). Separate samples (4 ml) were centrifuged at 2,000 × g for 20 min at 4°C using a Beckman J6-MI centrifuge (Beckman Coulter, Indianapolis, IN). Serum aliquots were collected and analyzed for serum biochemical components using a Roche Cobas 6000 c501 biochemistry analyzer (Roche Diagnostics, Indianapolis, IN).

### Body Composition Assessment

Body composition [FM, LBM, and total body water (TBW)] were measured using quantitative magnetic resonance (QMR) technology (CanCog Tech, Fergus, ON) on weeks −1 and 9. With the use of QMR technology, dogs were able to be imaged while avoiding anesthetization (30).

### Fecal Collection and Analysis

During fecal collection, whole samples were collected within 15 min of being voided, and all visible contaminants (e.g., grass and hair) and portions of samples that were in contact with the ground were removed. Samples were transferred into a Whirl-pak bag (Thermo Fisher Scientific, Waltham, MA) to be homogenized. Homogenized samples were then stored in 50-ml sterile centrifuge tubes (Thermo Fisher Scientific, Waltham, MA), frozen, and kept at −20°C until further analysis. Samples were analyzed for SCFAs (acetic acid, propionic acid, and butyric acid), branched-chain fatty acids (BCFAs; isobutyric acid and isovaleric acid), lactic acid, formic acid, and monosaccharides using an Agilent HP1000 series high-performance liquid chromatography (HPLC; Agilent Technologies, Santa Clara, CA).

HPLC analysis was based on a previous study from our laboratory by Templeman et al. (22). In brief, samples were prepared by combining 0.1 g of sample with 1 ml of 0.005 N sulfuric acid (concentrated sulfuric acid in Milli-Q water; Sigma Aldrich, Oakville, ON). Samples were vortexed until the fecal sample had completely dissolved and were then centrifuged using a Fisherbrand accuSpin Micro 17 (Thermo Fisher Scientific, Waltham, MA) at 13,300 rpm for 15 min. Four hundred microliters of supernatant was then drawn into an HPLC vial, and 400 μl of 0.005 M sulfuric acid was added to achieve a 2× dilution. For the HPLC system, the mobile phase was 0.005 M of sulfuric acid in Milli-Q water that was then filtered through a 0.2-μm filter. The column temperature was kept at 60°C, the refractive index detector temperature was kept at 40°C, and the injection volume was 20 μl. The flow rate was 0.5 ml/min, and the cycle time was 45 min. A standard curve was developed for each SCFAs, BCFAs, and monosaccharide with the following serial dilutions: 0.25, 0.50, 1.00, 2.00, and 4.00 mmol/L. OpenLAB CDS ChemStation software was used for system control and data acquisition (Agilent Technologies, Santa Clara, CA).

### Statistical Analyses

Statistical analyses were performed with Statistical Analysis System (SAS) (v. 9.4; SAS Institute Inc., Cary, NC). RR and BT data were analyzed for outliers by removing the data points that exceeded an RR <200 breaths/min and lower than 5 breaths/min. For BT, data points higher than 42°C and lower than 36°C were also considered outliers. Also, the periods during the exercise challenges when a four-dog team was stopped were removed, as these interruptions reduced the dogs' ability to maintain a wRR. These stops included instances of defecation and urination. Following data cleanup, a TRANSREG procedure was used to optimally transform the RR data. BT was calculated using the means of each 1-min interval from the start of each exercise challenge until 30 min post-exercise. Variances in RR, BT, fecal metabolites, fasted blood analytes (CBC and serum biochemistry), feed intake, BW, and body composition data were analyzed using PROC GLIMMIX of SAS (v. 9.4; SAS Institute Inc., Cary, NC). Dog was treated as a random effect; and activity level (resting, working, and post-exercise), week, and diet group (DG; Trt, or Ctl) were treated as fixed effects. Activity level was analyzed against diet group and week, as well as week against diet group. RR and fecal metabolite means were compared using the Tukey honestly significant difference (HSD), whereas BT means were compared using a Fishers least significant difference (LSD). Statistical significance was declared at *P* ≤ 0.05 and trends at 0.05 < *P* ≤ 0.10.

## Results

During data analysis, 18% (27/153) of the RR observations and 31% (433/1,378) of BT observations were removed due to either software malfunctions, which caused missing data, or movement of rectal probes during the challenge run. Two dogs were removed from the trial (one on week 2, Ctl; one on week 4, Ctl) due to exercise-related injuries; all data collected from these dogs until their removal were included in the results. Due to receiver limitations, only eight dogs were monitored for BT throughout the study (3 males; Trt = 1, Ctl = 2, 5 females; Trt = 3, Ctl = 2).

### Mean Daily Food Intake, Body Weight, and Body Composition

No differences were observed between mean daily feed intake, BW, and RD with diet (*P* > 0.10), but all variables differed by week (*P* < 0.05; Table 2). Feed intake was greatest at weeks 4–8 (*P* < 0.05; Table 2) with no differences between weeks 4 and 8 (*P* > 0.10; Table 2). Body weight decreased from baseline at weeks 2 and 3 (*P* < 0.05); however, BW did not differ from week −1 to weeks 0–1 (*P* > 0.10) and weeks 4–8 (*P* > 0.10; Table 2).

Body composition (FM, LBM, and TBW) at weeks −1 and 9 did not differ between diet groups (P > 0.10); however, when data were pooled to evaluate the effects of exercise, all variables differed by week (*P* < 0.05; Table 2). LBM increased by 7%, TBW increased by 5.3%, and FM decreased by 3.5% from week −1 to 9 (*P* < 0.05; Table 2).

### Body Temperature

No differences in rBT were observed between dietary treatments (*P* > 0.10; Table 3); however, Trt dogs presented with a lower mean wBT and post-BT than Ctl (*P* < 0.05, Table 3). Post-exercise BT was lower at weeks 2 and 5 for Trt vs. Ctl (*P* < 0.05; Table 3). No other differences were observed between DG; therefore, internal BT was pooled to evaluate the effect of exercise. No differences were observed between rBT over weeks (*P* > 0.10). Working BT tended to be lower at week 8 compared with week −1 (*P* < 0.10). Post-exercise BT was greater at week 5 compared with weeks 2 and 8 (*P* < 0.05) but was not different than week −1 (*P* > 0.10).

**Table 3:** Mean internal body temperature (°C) at resting, working, and post-exercise state for control (0.74% soluble fiber on a dry-matter basis) and treatment groups (2.12% soluble fiber on a dry-matter basis) in sled dogs running at a pace of 20 km/h for 3 km.

		**Exercise challenge**				**SEM (Wk)**	**Mean**	**SEM (mean)**	***P*****-value**		
		**Week −1**	**Week 2**	**Week 5**	**Week 8**				**DG^1^**	**Wk**	**DG × Wk^2^**
rBT^3^, °C	Trt	38.4	38.7	38.7	38.7	1.53	38.6	0.16	0.556	0.830	0.992
	Ctl	38.6	38.9	38.9	38.8	0.39	38.8	0.17			
wBT, °C	Trt	39.7^#^	39.2	39.7	39.3^#^	0.20	39.4^*^	0.14	0.006	0.159	0.108
	Ctl	39.8	39.9	39.8	39.6	0.19	39.8^*^	0.13			
post-BT, °C	Trt	39.6^b^	38.9^*^	39.3^a^^b^^*^	39.1^a^^b^	0.23	39.2^*^	0.13	0.009	0.055	0.012
	Ctl	39.3^b^	39.7^a^^b^^*^	40.1^a^^*^	39.3^b^	0.24	39.6^*^	0.13			

1 *DG, diet group; Trt, treatment dogs; Ctl, control dogs; SEM, standard error of the mean, n = 3 for rBT (Trt: n = 2; Ctl: n = 1, weeks −1 and 2), n = 7 for rBT (Trt: n = 4; Ctl: n = 3, week 5), and n = 4 for rBT (Trt: n = 3; Ctl: n = 1, week 8); n = 5 for wBT (Trt: n = 2; Ctl: n = 3, week −1), n = 7 for wBT (Trt: n = 3; Ctl: n = 4, week 2), n = 7 for wBT (Trt: n = 4; Ctl: n = 3, week 5), and n = 6 for wBT (Trt: n = 3; Ctl: n = 3, week 8); n = 5 for post-BT (Trt: n = 2,; Ctl: n = 3, week −1), n = 7 for post-BT (Trt: n = 3: Ctl: n = 4, week 2), n = 7 for post-BT (Trt: n = 4: Ctl: n = 3, week 5), and n = 6 for post-BT (Trt: n = 3: Ctl: n = 3, week 8)*.

2 *Interaction effect between DG and Wk (week)*.

3 *rBT, resting body temperature; wBT, working body temperature; post-BT, post-exercise body temperature*.

a, b *Values in a row with different superscripts differ (P < 0.05; within treatment)*.

# *Mean values for body temperature tended to differ from week −1 within the same row (P < 0.10)*.

* *Values for body temperature different between diet groups within the same column (P < 0.05)*.

### Respiratory Rate

No differences in rRR, wRR, and post-RR were observed between dietary treatments (*P* > 0.10; Table 4); therefore, these data were pooled to evaluate the effect of exercise. No differences in activity level (rRR, wRR, and post-RR) were observed between weeks throughout the study duration (*P* > 0.10; Table 4).

**Table 4:** Mean respiratory rate (breaths per minute) at rest, work, and post-exercise during weeks −1, 2, 5, and 8 for control (0.74% soluble fiber on a dry-matter basis) and treatment groups (2.12% soluble fiber on a dry-matter basis) in sled dogs running at a pace of 20 km/h for 3 km.

		**Exercise challenge**				**SEM (Wk)**	**Mean^a^**	**SEM (mean)**	***P*****-value**		
		**Wk −1**	**Wk 2**	**Wk 5**	**Wk 8**				**Wk**	**DG**	**DG × Wk^b^**
rRR^c^, bf^d^	Trt	18	16	17	18	1.53	17	0.60	0.481	0.262	0.790
	Ctl	19	18	17	18	1.71	18	0.67			
wRR, bf	Trt	173	171	170	172	2.89	172	2.69	0.934	0.143	0.354
	Ctl	165	166	166	165	3.39	165	2.96			
post-RR, bf	Trt	22	28	26	26	2.43	26	1.93	0.723	0.809	0.382
	Ctl	25	25	24	25	3.18	25	2.06			

a *Trt, treatment dogs; Ctl, control dogs; SEM, standard error of the mean, n = 11 for rRR (week −1), n = 13 for rRR (week 2), and n = 10 for rRR (weeks 5 and 8); n = 13 for wRR (week −1), n = 11 for wRR (week 2), and n = 10 for wRR (weeks 5 and 8); n = 9 for post-RR (weeks −1, 2, and 5), and n = 11 for post-RR (week 8)*.

b *Interaction effect between DG and Wk (week)*.

c *rRR, resting respiratory rate; wRR, working respiratory rate; post-RR, post-exercise respiratory rate*.

d *bf, breathing frequency (breaths per minute)*.

### Fecal Short-Chain Fatty Acids, Branched-Chain Fatty Acids, Lactic Acid, and Monosaccharides

Overall, dogs receiving the Trt diet had greater concentrations of fecal propionic acid (*P* < 0.05) and butyric acid (*P* < 0.05; Table 5) than Ctl. Concentrations of fecal acetic acid tended to be greater in Trt compared with Ctl (*P* = 0.06; Table 5). Treatment dogs had greater fecal isobutyric acid concentrations than Ctl (*P* < 0.05; Table 5). In addition, Trt dogs had greater fecal concentrations of lactose, glucose, xylose, and arabinose than Ctl (*P* < 0.05; Table 5). Diet had no effect on fecal formic acid, isovaleric acid, or lactic acid (*P* > 0.10; Table 5); therefore, all remaining data were pooled for effect of exercise. For all dogs, fecal propionic acid concentrations were greater during weeks 0–8 than week −1 (*P* < 0.05; Table 5). Fecal butyric acid concentrations were greater during weeks 7 and 8 compared with week −1 (*P* < 0.05; Table 5). Fecal acetic acid concentrations were greater during weeks 2–8 compared with week −1 and greater at week 8 compared with week 0 (*P* < 0.05; Table 5). Week had no effect on fecal formic acid concentrations (*P* > 0.10; Table 5). Fecal isobutyric acid was greater at week −1 compared with weeks 0–2 (*P* < 0.05; Table 5) but did not differ thereafter (*P* > 0.10; Table 5). Week had no effect on fecal isovaleric acid concentrations (*P* > 0.10; Table 5). Fecal lactic acid concentration was greater during weeks 3 and 5 compared with week 7 (*P* < 0.05), but no other weeks differed (*P* > 0.10; Table 5). Fecal lactose concentrations were greater in week −1 than weeks 5, 7, and 8 (*P* < 0.05; Table 5). Fecal glucose concentrations were greater in week 0 compared with week 2 (*P* < 0.05; Table 5). Week had no effect on fecal xylose and arabinose concentrations (*P* > 0.10; Table 5).

**Table 5:** Fecal concentrations of short-chain fatty acids, branched-chain fatty acids, lactic acid, and monosaccharides of sled dogs being fed a diet containing a soluble fiber content of 0.74% (Ctl) or 2.12% (Trt) throughout 9 weeks of incremental exercise conditioning.

	**Week**										**SEM^1^**	**Diet**		**SEM**	***P*****-value**		
	**−1**	**0**	**1**	**2**	**3**	**4**	**5**	**6**	**7**	**8**		**Trt**	**Ctl**		**Wk**	**DG**	**DG × Wk^2^**
Acetic acid^3^	75.10^c^	86.22^b^^c^	89.28^b^^a^^c^	95.23^b^^a^	102.00^b^^a^	98.30^b^^a^	95.75^b^^a^	96.52^b^^a^	102.83^b^^a^	108.21^a^	5.12	100.53	89.36	5.41	<0.01	0.06	0.29
Propionic acid	20.04^b^	30.59^a^	29.52^a^	31.26^a^	29.46^a^	28.93^a^	20.34^a^	32.18^a^	34.34^a^	36.48^a^	3.03	35.55^*^	25.08	1.51	<0.01	<0.01	0.02
Butyric acid	18.40^b^^c^^d^	18.09^abcd^	15.66^d^	17.94^c^^d^	22.54^a^^b^	22.54^a^^b^	23.28^a^^b^	22.37^a^^b^^c^	25.22^a^	25.54^a^	2.00	22.76^*^	19.56	0.89	<0.01	<0.01	0.58
Formic acid^4^	2.22	2.20	2.26	2.22	2.22	2.23	2.23	2.20	n/a^5^	2.22	0.04	2.22	2.22	0.02	0.30	0.81	0.25
Isobutyric acid	17.15^a^	12.91^b^	12.66^b^	11.92^b^	19.71^a^	19.99^a^	19.39^a^	21.14^a^	21.85^a^	20.64^a^	1.45	18.58^*^	16.90	0.69	<0.01	0.02	0.15
Isovaleric acid	5.49	5.35	5.49	5.69	5.51	5.88	5.91	5.68	6.61	5.71	0.49	5.66	5.81	0.32	0.11	0.65	0.26
Lactic acid	2.19^a^^b^	2.17^a^^b^	2.20^a^^b^	2.23^a^^b^	2.23^a^	2.23^a^^b^	2.25^a^	2.19^a^^b^	2.14^b^	2.16^a^^b^	0.03	2.21	2.19	0.02	<0.01	0.52	0.70
Lactose	4.63^b^	5.11^a^^b^	5.69^a^^b^	6.08^a^^b^	5.99^a^^b^	6.57^a^^b^	7.22^a^	6.94^a^^b^	7.70^a^	8.08^a^	0.75	7.07^*^	5.73	0.51	0.01	0.01	0.28
Glucose	1.96^a^^b^	2.01^a^	1.96^a^^b^	1.91^b^	1.93^a^^b^	1.92^a^^b^	2.02^a^^b^	n/a	n/a	1.96^a^^b^	0.04	1.99^*^	1.93	0.02	0.02	0.01	0.02
Xylose	4.28	3.56	3.26	3.24	3.30	3.54	4.24	3.15	3.25	2.83	0.79	4.25^*^	2.68	0.28	0.22	<0.01	0.09
Arabinose	5.28	5.22	4.67	3.97	3.68	3.61	6.49	4.01	4.63	3.67^a^	0.94	5.03^*^	4.03	0.39	0.07	0.01	0.08

1 *Standard error of the mean*.

2 *Interaction effect between DG and Wk (week)*.

3 *Data presented in micromoles per gram (μmol/g) on a dry-matter basis; SCFA: acetic acid + propionic acid + butyric acid; BCFA: isobutyric acid + isovaleric acid; monosaccharides: lactose, xylose, arabinose*.

4 *Data presented in nanomoles per gram (nmol/g) on a dry-matter basis; formic acid; lactic acid; glucose*.

abcd *Values in a row (within week) with different superscripts differ (P ≤ 0.05)*.

* *Asterisk indicates difference between diet groups (P ≤ 0.05)*.

*^N/A^Insufficient data*.

### Complete Blood Count and Serum Biochemistry

Treatment dogs had greater concentrations of white blood cells, leukocytes (Supplementary Table 1), and alanine aminotransferase (ALT; *P* < 0.05; Supplementary Table 2) and lower concentrations of creatinine (*P* < 0.05; Supplementary Table 2) than Ctl dogs; however, all mean CBC and serum biochemistry values were within standard reference range for dogs of both diet groups (as determined by the Animal Health Laboratory, University of Guelph, Guelph, ON). Except for five dogs that presented with high eosinophils at weeks −1 and 2, all dogs remained healthy throughout the study period (Supplementary Table 1).

## Discussion

This study was the first to evaluate the effects of increased dietary soluble fiber on internal BT, RR, body composition, and fecal metabolites in mid-distance training sled dogs. The data presented herein indicate that for actively training sled dogs, 9 weeks of an increased soluble fiber diet may have contributed to a decrease in the working and recovery state internal BT. In addition, dogs receiving the soluble fiber-supplemented diet had greater fecal concentrations of butyric and propionic acid than had Ctl dogs; however, increased soluble fiber had no effect on RR or body composition. Participation in 9 weeks of incremental exercise resulted in an overall increase in LBM and decrease in FM.

### Body Temperature

Given that dogs primarily dissipate heat through evaporative panting (31), water lost by way of salivation increases considerably during exercise (32), contributing to an increased risk of dehydration, which, in turn, may negatively impact thermoregulatory capabilities (32). For example, sedentary dogs dehydrated by way of water restriction or induced hypertonic dehydration have been reported to have elevated BT when compared to hydrated dogs (14, 16, 33). As the Trt dogs in the current study presented with an overall reduction in internal temperature at both a working and post-exercise state when compared to the Ctl dogs, this suggests that supplementation of soluble fiber so as to achieve an inclusion level of 2.12% may have contributed to a more efficient regulation of internal BT during exercise. For example, exercising horses fed diets high in soluble fiber were reported to have greater TBW and lower exercising BT than horses fed insoluble fiber (34); however, it should be noted that unlike dogs, horses dissipate heat primarily through active sweating (35). As the diet in the current study had no impact on changes in TBW content, and since no other measurements of hydration status were reported, we cannot definitely say that increased dietary soluble fiber improved hydration status; we can only make inferences based on changes in BT. In addition, as only eight dogs were evaluated for changes in BT, the authors acknowledge this as a limitation. Therefore, future research is warranted to investigate hydration status, exercise, and internal BT using a greater sample size of actively training dogs to support these pilot data.

Independent of diet, conditioning has been reported to reduce exercising BT in both humans (36, 37) and dogs (38). In the current study, wBT at week 8 tended to decrease from baseline, suggesting a possible improvement in the thermoregulatory ability as training progressed. As exercise training leads to an increased cardiac output with increased blood volumes directed toward respiratory muscles (39), the improved regulation of BT is thought to be due to the increased blood flow to areas of heat exchange supporting thermoregulation in dogs (40). Therefore, as previous studies report improved cardiorespiratory capacity with exercise (41, 42), the likelihood of such an event occurring in the current study is high. However, as only RR was measured in the current study, with no other cardiorespiratory variables, the ability to relate thermoregulation to improved exercise conditioning in exercising dogs requires further investigation. However, as exercise usually takes place in colder environments, this reduction in BT could also be attributed to ambient temperature. However, as both BT and ambient temperature were highly variable throughout the current training period, future research should be conducted to investigate the association between ambient temperature and internal BT in dogs exercising in cold climates.

### Respiratory Rate

Hydration status has been reported to influence RR, as dehydrated dogs have lower RR (14) in an attempt to conserve body water content while panting (15, 33). Although the current study reported no changes in RR following an increased soluble fiber diet, the level of dehydration during exercise may not have been great enough to influence RR. For example, a previous study from our laboratory reported that sled dogs running 5 km daily led to signs of hypertonic dehydration (13), whereas the dogs in the current study were running just over 3 km daily. This suggests that unlike BT, RR may not be affected by slight changes in hydration. Future research is warranted to investigate changes in hydration status with a 2.12% soluble fiber diet in relation to RR in both a sedentary and exercising dog model.

For all dogs, 9 weeks of endurance exercise training had no effect on RR during any given activity level. Our laboratory previously reported that 12 weeks of aerobic exercise training resulted in a decreased RR at both a resting and post-exercise state with changes starting at week 5 (29). As the dogs in the current study were running 25.4 km/day during week 5 and the previous study's RD was 37.2 km/day at week 5, this 11.8 km/day difference could be behind the lack of exercise effects. In addition, the current study began descaling the training regimen at week 5, whereas Thornton et al. (29) began descaling at week 7. As a result, the changes in duration could have influenced RR, as our lab previously reported that RR may be sensitive to a training regimen and requires a continuous incremental training regimen to elicit changes (29).

### Body Composition

Participation in regular aerobic exercise positively affects body composition resulting in reductions in FM and increases in LBM in humans (20, 43). In dogs, 12 weeks of endurance training resulted in increased LBM of 11% and decreased FM of 4.5% (22). The current study reported an increase in LBM of 7% and FM decrease of 3.5%, with diet having no effect on changes in body composition. As previous studies examined the effects of fiber supplementation on body composition utilizing overweight, sedentary subjects (19), the effects of exercise in the current study may have been greater than those of increased soluble fiber. For example, as BW was maintained and feed intake was adjusted to maintain energy requirements for increased exercise, the changes seen in LBM and reduction in FM can likely be attributed to the exercise regimen itself.

### Fecal Metabolites

Soluble fiber is known to increase microbial fermentation and production of SCFAs, which can have a variety of physiological effects. For example, SCFAs can stimulate an increase in water absorption, as these major anions have been reported to be responsible for osmotic water absorption in the colon of the dogs (12). As Trt dogs in the current study had greater concentrations of both propionic and butyric acid than had Ctl dogs, there is an increased likelihood of greater water retention in the dogs consuming the higher soluble fiber diet. As such, this could lead to possible secondary effects in the dogs, resulting in lower internal BT, which could in turn help with exercise performance. Additionally, as exercise duration increased over time, the dogs' energy requirements also increased, leading to greater feed intake and subsequently protein intake. Fecal isobutyric acid concentration was greater in Trt dogs and has been reported in dogs consuming greater levels of dietary protein (44). However, as protein contents of both diets were similar and overall feed intake did not differ between treatment groups, it is possible that the inclusion of soluble fiber reduced the digestibility of protein, thereby increasing its availability for fermentation in the colon (45). Together, because SCFAs, BCFAs, and other fecal metabolites increased over time, it is likely that the environment of the hindgut microbiota was altered. As sled dogs are at risk of impaired gut health (i.e., increased gut permeability) leading to instances of diarrhea and other deleterious outcomes, provision of soluble fiber can support inflammatory responses and reduce exercise-induced diarrhea (46, 47). As diarrhea is the leading cause of exercise discontinuation in exercising sled dogs (1, 2), minimizing pathogenic microbes could have beneficial outcomes related to performance. For example, sled dogs running in a 400-km race in Norway presenting with low levels of dysbiosis-associated bacteria (i.e., *Fusobacterium, Clostridium hiranonis*, and *Blautia*) prior to a race performed better than dogs with a greater level of these bacteria (48). As such, the fecal metabolite data in the current study suggest a shift toward an improved gut microbial environment. However, due to restrictions of laboratory analysis during 2020, the effects of both exercise and dietary soluble fiber on the gut microbiota were not able to be assessed. Therefore, future research should be directed toward evaluating microbial diversity and stool quality during exercise and nutritional interventions in training sled dogs.

## Conclusion

The findings in the current study suggest that the addition of 1.38% soluble fiber to achieve an insoluble:soluble fiber ratio of 3:1 may have contributed to a reduction in internal BT at both working and recovery post-exercise states and resulted in greater fecal SCFA concentrations. Supplemental soluble fiber had no effect on RR or body composition; however, a 9-week training regimen resulted in increased LBM and decreased FM. Future research is warranted to investigate how a diet with a soluble fiber inclusion of 2.12% influences hydration status, and various cardiorespiratory measurements (i.e., heart rate and VO_2_max) in exercising dogs. Overall, these results can be used to improve training regimens and diets that may influence exercise physiology, health, and performance of sled dogs.

## Data Availability Statement

The original contributions presented in the study are included in the article/Supplementary Material, further inquiries can be directed to the corresponding author/s.

## Ethics Statement

The animal study was reviewed and approved by University of Guelph's Animal Care Committee. Written informed consent was obtained from the owners for the participation of their animals in this study.

## Author Contributions

ET, JT, KS, and AS designed the experiment. ET, ER, and JT conducted the research. ET and LB analyzed the data. AS had primary responsibility for the final content. All authors read and approved the final manuscript. All authors contributed to the writing of the manuscript.

## Conflict of Interest

MB was employed by the company emka Technologies. The remaining authors declare that the research was conducted in the absence of any commercial or financial relationships that could be construed as a potential conflict of interest. The authors declare that this study received funding from Champion Petfoods Ltd. The funder was not involved in the study design, collection, analysis, interpretation of data, the writing of this article or the decision to submit it for publication.
