# Supplementary material for: Supplemental Fiber Affects Body Temperature and Fecal Metabolites but Not Respiratory Rate or Body Composition in Mid-Distance Training Sled Dogs
Source: Front Vet Sci. 2021 Apr 29;8:639335. doi: 10.3389/fvets.2021.639335 (PMC8116954; doi:10.3389/fvets.2021.639335)
Supplement: Supplementary file 1 [file Data_Sheet_1.pdf]

Supplementary Table 1. Complete blood count values at weeks -1, 2, 5, and 8 for all dogs, and mean values (±SEM[1]) across all weeks for control or increased soluble fiber (treatment) dogs taken at rest.

Parameter[2]	Reference range	Week				Diet group		P-value		
		-1	2	5	8	Trt[3]	Ctl	DG	Wk	DG x Wk[4]
WBC, x10^9/L	4.9-15.4	11.40 ± 0.69	12.00 ± 0.69	11.96 ± 0.69	10.56 ± 0.50	12.74 ± 0.75*	10.23 ± 0.75	0.03	0.15	0.38
RBC, x10^12/L	5.8-8.5	7.36 ± 0.15[a]	7.31 ± 0.15[a]	6.69 ± 0.15[b]	7.12 ± 0.15[a]	7.17 ± 0.15	7.07 ± 0.15	0.65	≤0.01	0.79
HGB, g/L	133-197	174.86 ± 3.54[a]	172.29 ± 3.54[a]	158.34 ± 3.54[b]	167.36 ± 3.54[a]	167.07 ± 3.44	169.35± 3.44	0.64	≤0.01	0.80
HCT, L/L	0.39-0.56	0.51 ± 0.01[a]	0.50 ± 0.01[ab]	0.44 ± 0.01[c]	0.47 ± 0.01[d]	0.49 ± 0.01	0.48 ±0.01	0.46	≤0.01	0.85
MCV, fL	66-75	69.64 ± 0.65[a]	68.78 ± 0.65[a]	**65.87** ± 0.65[b]	66.32 ± 0.65[b]	67.50 ± 0.57	67.81 ± 0.59	0.71	≤0.01	0.15
MCH, pg	21-25	23.71 ± 0.19	23.64 ± 0.19	23.79 ± 0.19	23.57 ± 0.19	23.68 ± 0.23	23.68 ± 0.23	0.98	0.41	0.72
MCHC, g/L	321-360	341.71 ± 2.49[b]	342.93 ± 2.49[b]	360.30 ± 2.60[a]	354.93 ± 2.74[a]	349.58 ± 1.67	350.36 ± 1.53	0.74	≤0.01	0.07
RDW, %	11-14	12.31 ± 0.20	12.41 ± 0.20	12.07 ± 0.20	12.08 ± 0.20	12.22 ± 0.18	12.21 ± 0.18	0.98	0.42	0.05
PLTS, x10^9/L	117-418	276.57 ± 25.6[a]	275.93 ± 25.6[a]	317.25 ± 25.6[b]	308.44 ± 25.6[b]	295.54 ± 33.58	293 ± 33.58	0.97	≤0.01	0.84
MPV, fL	7-14	9.46 ± 0.20[b]	9.96 ± 0.20[a]	9.31 ± 0.20[b]	9.44 ± 0.20[b]	9.46 ± 0.25	9.63 ± 0.25	0.62	≤0.01	0.60
PLTCRT, %	0.14-0.47	0.26 ± 0.02[a]	0.27 ± 0.02[a]	0.29 ± 0.02[a]	0.29 ± 0.02[a]	0.27 ± 0.03	0.28 ± 0.03	0.87	0.05	0.77
TSP, g/L	55-75	75.14 ± 0.72[a]	70.71 ± 0.72[bc]	72.18 ± 0.72[b]	68.81 ± 0.72[c]	73.6 ± 0.58	71.36 ± 0.58	0.15	≤0.01	0.66
NCT, x10^9/L	2.9-10.6	6.17 ± 0.48[b]	6.65 ± 0.48[ab]	7.18 ± 0.48[a]	5.87 ± 0.48[b]	7.08 ± 0.55	5.86 ± 0.55	0.14	≤0.05	0.17
LCT, x10^9/L	0.8-5.1	2.97 ± 0.23	3.21 ± 0.23	2.84 ± 0.23	2.91 ± 0.23	3.47 ± 0.27*	2.49 ± 0.27	0.02	0.32	0.37
MCT, x10^9/L	0-1.1	0.59 ± 0.09	0.67 ± 0.09	0.76 ± 0.09	0.71 ± 0.09	0.78 ± 0.08	0.58± 0.08	0.10	0.50	0.09
ECT, x10^9/L	0.08-1.33	**1.63** ± 0.27	**1.45** ± 0.27	1.04 ± 0.29	1.04 ± 0.29	1.36 ± 0.27	1.28 ± 0.28	0.85	0.29	0.37

[1]Standard error of the mean, n = 15 for wks -1 to 6 and n = 13 for wks 9 and 11, n = 8 for treatment diet group, n = 7 for control diet group.
[2]WBC, white blood cell; RBC, red blood cell; HGB, hemoglobin; HCT, hematocrit; MCV, mean corpuscular volume; MCH, mean corpuscular hemoglobin; MCHC mean corpuscular hemoglobin concentration; RDW, red cell distribution width; PLTS, platelets; MPV, mean platelet volume; PLTCRT, plateletcrit; TSP, total serum protein; NCT, neutrophil count; LCT, lymphocyte count; MCT, monocyte count; ECT, eosinophil count.
[3]Trt, treatment; Ctl, control; DG, diet group; Wk, Week.
[4]Interaction effect between diet group and wk.
[a,b,c] Values in a row with different superscript are different ($P \leq 0.05$).

*Mean value for treatment dogs significantly differs from control dogs within the same row ($P \leq 0.05$).
Bolded values indicate complete blood count values that fall outside of standard reference range (as determined by Animal Health Laboratories, University of Guelph, Guelph, ON.

Supplementary Table 2. Serum biochemistry values at weeks -1, 2, 5, and 8 for all dogs, and mean values (±SEM[1]) across all weeks for control or increased soluble fiber (treatment) dogs taken at rest.

**Parameter**[2]	**Reference range**	**Week**				**Diet group**		**P-value**		
		-1	2	5	8	Trt[3]	Ctl	DG	Wk	DG x Wk[4]
Ca, mmol/L	2.5-3	**2.29** ± 0.09	**2.44** ± 0.09	**2.44** ± 0.09	**2.46** ± 0.09	2.35 ± 0.07	2.47 ± 0.07	0.26	0.53	0.45
P, mmol/L	0.9-1.85	1.28 ± 0.04[a]	1.26 ± 0.04[ac]	1.18 ± 0.04[b]	1.20 ± 0.034[bc]	1.24 ± 0.05	1.23 ± 0.05	0.96	≤0.05	0.83
Mg, mmol/L	0.7-1	0.83 ± 0.02[a]	0.84 ± 0.02[ab]	0.76 ± 0.02[c]	0.80 ± 0.02[d]	0.81 ± 0.02	0.81 ± 0.02	0.92	≤0.01	0.39
Na, mmol/L	140-154	148.20 ± 0.42[c]	146.77 ± 0.42[a]	145.48 ± 0.42[b]	146.48 ± 0.42[ab]	146.75 ± 0.36	146.72 ± 0.35	0.95	≤0.01	0.59
K, mmol/L	3.8-5.4	4.81 ± 0.06[bc]	5.03 ± 0.06[a]	4.71 ± 0.06[b]	4.89 ± 0.06[ac]	4.79 ± 0.05	4.92 ± 0.05	0.10	≤0.01	0.27
Cl, mmol/L	104-119	112.86 ± 0.41[a]	113.70 ± 0.41[a]	111.79 ± 0.41[b]	111.34 ± 0.41[b]	112.29 ± 0.35	112.56 ± 0.35	0.59	≤0.01	0.08
CO_2, mmol/L	15-25	17.40 ± 0.65	17.01 ± 0.65	15.93 ± 0.65	17.33 ± 0.65	17.04 ± 0.51	16.79 ± 0.51	0.73	0.28	0.50
Anion gap, mmol/L	13-24	22.81 ± 0.44[a]	22.11 ± 0.44[b]	22.66 ± 0.44[a]	22.75 ± 0.44[a]	22.42 ± 0.43	22.25 ± 0.43	0.79	≤0.01	0.38
Na: K	29-37	30.86 ± 0.37[ab]	29.38 ± 0.37[b]	30.93 ± 0.37[ac]	29.80 ± 0.37[bc]	30.71 ± 0.33	29.77 ± 0.33	0.06	≤0.01	0.25
TP, g/L	55-74	62.32 ± 0.67[a]	61.61 ± 0.67[ab]	60.09 ± 0.67[b]	61.43 ± 0.67[ab]	61.89 ± 0.68	60.83 ± 0.68	0.29	≤0.05	0.88
Albumin, g/L	29-43	38.99 ± 0.54[a]	36.95 ± 0.54[b]	37.59 ± 0.54[ab]	37.72 ± 0.54[ab]	38.21 ± 0.57	37.41 ± 0.57	0.34	≤0.01	0.34
Globulin, g/L	21-42	23.33 ± 0.68[ab]	24.66 ± 0.68[a]	22.49 ± 0.68[b]	23.70 ± 0.68[ab]	23.68 ± 0.71	23.42 ± 0.71	0.79	≤0.05	0.78
A: G	0.7-1.8	1.69 ± 0.06[a]	1.52 ± 0.06[b]	1.69 ± 0.06[a]	1.60 ± 0.06[ab]	1.63 ± 0.06	1.62 ± 0.06	0.96	≤0.05	0.49
Urea, mmol/L	3.5-10	7.01 ± 0.40[c]	8.48 ± 0.40[b]	**10.48** ± 0.40[a]	**10.34** ± 0.40[a]	8.72 ± 0.37	9.44 ± 0.37	0.19	≤0.01	0.66
Creatinine, µmol/L	20-150	64.59 ± 3.28	66.94 ± 3.28	63.21 ± 3.28	66.06 ± 3.28	60.22 ± 2.45*	70.17 ± 2.45	0.01	0.82	0.23
Glucose, mmol/L	3.3-7.3	5.68 ± 0.11	5.41 ± 0.11	5.68 ± 0.11	5.36 ± 0.11	5.50 ± 0.08	5.55 ± 0.08	0.67	0.05	0.81
Cholesterol, mmol/L	3.6-10.2	4.19 ± 0.22[b]	4.65 ± 0.22[bc]	5.13 ± 0.22[ac]	5.01 ± 0.22[ac]	4.65 ± 0.27	4.84 ± 0.27	0.63	≤0.01	0.06
TB, µmol/L	0-4	1.07 ± 0.16	1.34 ± 0.16	0.97 ± 0.16	1.16 ± 0.16	1.25 ± 0.14	1.02 ± 0.14	0.27	0.27	0.64
CB, µmol/L	0-1	0.36 ± 0.14[b]	0.65 ± 0.14[ab]	0.99 ± 0.14[a]	0.60 ± 0.14[ab]	0.68 ± 0.08	0.62 ± 0.09	0.67	≤0.05	0.69

FB, µmol/L	0-3	0.71 ± 0.18^{a}	0.71 ± 0.18^{a}	0.0 ± 0.18^{b}	0.59 ± 0.18^{ab}	0.57 ± 0.13	0.43 ± 0.13	0.47	≤0.05	0.86
ALP, U/L	22-143	24.73 ± 6.53^{b}	25.87 ± 6.53^{ab}	35.11 ± 6.53^{ab}	39.31 ± 6.53^{a}	38.52 ± 7.66	24.00 ± 8.03	0.21	≤0.05	0.48
SIALP, U/L	0-84	9.89 ± 7.12	11.41 ± 7.12	19.77 ± 7.12	20.16 ± 7.12	6.59 ± 9.11	24.02 ± 9.11	0.19	0.10	0.35
GCT, U/L	0-7	0.73 ± 0.35^{c}	1.78 ± 0.35^{ab}	2.45 ± 0.35^{a}	1.24 ± 0.35^{bc}	1.54 ± 0.33	1.56 ± 0.33	0.95	≤0.01	0.24
ALT, U/L	19-107	29.43 ± 5.35	33.85 ± 5.35	37.74 ± 5.35	35.84 ± 5.35	42.57 ± 4.91*	25.86 ± 4.91	0.03	0.55	0.06
CK, U/L	40-255	106.22 ± 26.61	159.72 ± 26.61	102.22 ± 26.61	107.52 ± 26.61	122.43 ±20.18	115.41 ± 20.18	0.80	0.27	0.99
Amylase, U/L	299-947	348.03 ± 25.09^{a}	318.46 ± 25.09^{ab}	**284.31** ± 25.09^{b}	**272.21** ± 25.09^{b}	291.07 ± 28.93	320.44 ± 28.93	0.47	≤0.01	0.25
Lipase, U/L	25-353	80.43 ± 6.06	77.45 ± 6.06	73.68 ± 6.06	75.01 ± 6.06	84.89 ± 7.33	68.39 ± 7.33	0.13	0.53	0.60

[1]Standard error of the mean, n = 15 for wks -1 to 6 and n = 13 for wks 9 and 11, n = 8 for treatment diet group, n = 7 for control diet group.

[2]Ca, calcium; P, phosphorus; Mg, magnesium; Na, sodium; K, potassium; Cl, chloride; CO_2, carbon dioxide; Na: K, sodium to potassium ratio; TP, total protein; A: G, albumin to globulin ratio; TB, total bilirubin; CB, conjugated bilirubin; FB, free bilirubin; ALP, alkaline phosphatase; SIALP, steroid induced alkaline phosphatase; GCT, glucose challenge test; ALT, alanine transaminase; CK, creatinine kinase.

[3]Trt, treatment; Ctl, control; DG, diet group.

[4]Interaction effect between diet group and wk (week).

[a,b,c] Values in a row with different superscript are different ($P \leq 0.05$).

*Mean value for treatment dogs significantly differs from control dogs within the same row ($P \leq 0.05$).

Bolded values indicate serum biochemistry values that fall outside of standard reference range (as determined by Animal Health Laboratories, University of Guelph, Guelph, ON.
